# Prognostic significance and optimal cutoff of age in medullary thyroid cancer

**DOI:** 10.18632/oncotarget.7556

**Published:** 2016-02-21

**Authors:** Ning Qu, Rong-liang Shi, Ting-xian Luo, Yu-long Wang, Duan-shu Li, Yu Wang, Cai-ping Huang, Qing-hai Ji

**Affiliations:** ^1^ Department of Head and Neck Surgery, Fudan University Shanghai Cancer Center, Department of Oncology, Shanghai Medical College, Fudan University, Shanghai, China; ^2^ Department of General Surgery, Minhang Hospital, Fudan University, Shanghai, China

**Keywords:** medullary thyroid cancer, age, prognosis, SEER, nomogram

## Abstract

Age has been found to correlate with the prognosis for medullary thyroid cancer (MTC). This study was conducted to investigate whether age can predict long-term unfavorable prognosis and evaluate its predictive accuracy associated with TNM staging, using data of patients diagnosed with MTC between 2000 and 2010 from Surveillance, Epidemiology and End Results database. The relationship between the patients’ age at diagnosis and cancer-specific survival (CSS) was evaluated using multivariate Cox regression analysis. Age stratifications were combined into a nomogram model to predict the CSS of MTC. The X-tile program determined 49 and 69 as optimal age cutoff values for CSS. On multivariate analysis, independent factors for survival were age (50–69 years, HR 2.853, 95% CI 1.631–4.991; ≥70 years, HR 5.804, 95% CI 2.91–11.555), race (white, HR 0.344, 95% CI 0.188–0.630), T (T3/4, HR 3.931, 95% CI 2.093–7.381), N (N1a, HR 3.269, 95% CI 1.386–7.710) and M (M1, HR 3.998, 95% CI 2.419–6.606). The C-index for CSS prediction with TNM, age (cutoff of 45)/sex/race/TNM and age (cutoff of 49 and 69)/sex/race/TNM were 0.832 (95% CI 0.763–0.901), 0.863 (95% CI 0.799–0.928), and 0.876 (95% CI 0.817–0.935), respectively. Subgroup multivariate analyses also showed that age significantly increased the risk for CSS in females, non-Hispanic white patients, and those with stage IV MTC. In conclusion, CSS was independently associated with ages between 49 and 69 years, which might be applied for risk stratification in MTC patients.

## INTRODUCTION

Thyroid cancer (TC) is the most common endocrine malignancy. With the annually increasing incidence, approximately 62,980 estimated cases of TC were newly diagnosed in the United States in 2014 [1]. Medullary thyroid carcinoma (MTC) is a rare malignancy that arises from the parafollicular or C-cells of neuroendocrine origin. It accounts for less than 10% of all thyroid cancers [2]. It occurs in hereditary (25% of cases) and sporadic (75%) forms; the latter is accompanied with 40% to 50% somatic mutations or rearrangements involving RET as reported in a previous study [3]. The prognosis for TC is known to be excellent; unfortunately, prognosis after treatment is unsatisfactory in MTC when compared with differentiated thyroid cancer (DTC) [4-6]. The carcinogenesis and biological behaviors of MTC differ from those of DTC (papillary thyroid cancer, follicular thyroid cancer). Further, MTC is frequently accompanied by aggressive locoregional diseases or distant metastases at the time of diagnosis [7-9]. Therefore, MTC is a distinct thyroid malignancy that requires a distinct prognostic predictive model.

Age has been shown to be such a strong prognostic factor of TC that it features prominently in the staging protocol of the American Joint Committee on Cancer (AJCC). In that protocol, age is the only determinant of stage for DTC rather than MTC, which seems to be a unique characteristic among all neoplasms. However, in a more recent review by Rendel *et al.* it was confirmed that the most sensitive predictors of survival in MTC patients were age at diagnosis and tumor stage [10].

In an effort to elucidate the prognostic value of both age and conventional factors under the current AJCC staging protocol, we sought to examine the survival trends of patients with MTC as reported by the Surveillance, Epidemiology, and End Results (SEER) Program from 2000 to 2010. In particular, we compared the survival of patients after stratification by age with that of conventional factors, aiming to determine whether age alone could improve mortality prediction to warrant the classification of AJCC staging. Nomograms are statistical tools shown to accurately and individually predict the outcomes of patients by including multiple variables in addition to the standard variables. Such standard variables are type of tumor (T), lymph node involvement (N), and presence of distant metastasis (M) of the TNM staging system, which have been widely accepted for the systematic evaluation of various cancers [11]. Therefore, we created the MTC nomogram based on patient age to accurately predict cancer-specific survival (CSS). The expectation of the present study was that age could be proposed as a supplemental element for determining prognosis and the intensity of follow-up in postoperative MTC patients.

## RESULTS

### Baseline characteristics and identification of metastatic lymph node ratio cutoff points

The total of 726 eligible patients consisted of 303 males and 423 females, with a median (range) age of 50 (4-89) years during the 10-year study period. Of these patients, 682 (93.9%) had total thyroidectomy (TT). The final pathology results revealed that there were 342 patients (47.1%) with MTC stage I, 81 (11.2%) with stage II, 74 (10.2%) with stage III, and 229 (31.5%) with stage IV. The clinicopathological characteristics of all patients are summarized in Table 1.

**Table 1:** Clinicopathological characteristics of 726 MTC patients from the SEER database

Variables	Total (%)
Sex	
Male	303 (41.7)
Female	423 (58.3)
Age	49.3 ± 16.1 (4–89)
Race	
Black	66 (9.1)
white	627 (86.4)
Other^1^	33 (4.5)
T stage	
T1/T2	445 (61.3)
T3/T4	281 (38.7)
N stage	
N0	320 (44.1)
N1a	113 (15.6)
N1b	293 (40.4)
M stage	
M0	627 (86.4)
M1	99 (13.6)
TNM stage	
I	342 (47.1)
II	81 (11.2)
III	74 (10.2)
IV	229 (31.5)
Treatment	
TT or near TT	682 (93.9)
Radioisotopes	22 (3.0)
Beam radiation	107 (14.7)

Data are presented as n (%) or mean ± standard deviation (range).

1 Includes American Indian/AK Native, Asian/Pacific Islander.

Abbreviations: MTC, medullary thyroid cancer; SEER, Surveillance, Epidemiology, and End Results; TT, total thyroidectomy.

**Figure 1:**
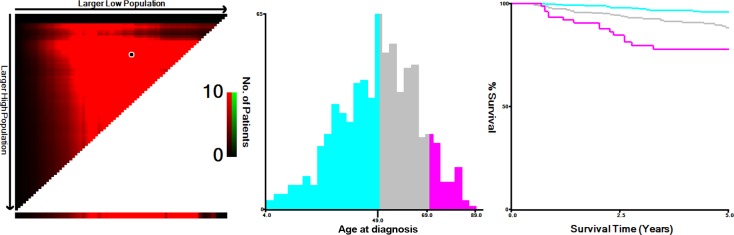
X-tile analysis of survival data from the SEER registry X-tile analysis was done on patient data from the SEER registry, equally divided into training and validation sets. X-tile plots of training sets are shown in the left panels, with plots of matched validation sets shown in the smaller inset. The optimal cut-point highlighted by the black circle in the left panels is shown on a histogram of the entire cohort (middle panels) and a Kaplan-Meier plot (right panels). *P* values were determined by using the cut-point defined in the training set and applying it to the validation set. Figures show age divided at the optimal cut-point (49 and 69, χ^2^ = 29.912, *p* < 0.001).

The mean follow-up duration was 58 (range 6-147) months. The overall CSS was 90.8% at 5 years and 81.2% at 10 years in this cohort of MTC patients. X-tile plots were constructed and the maximum of χ^2^ log-rank values of 29.912 was produced applying the ages 49 and 69 years as cutoff values to divide the cohort into high, middle, and low subsets in terms of CSS (Figure 1).

### Comparisons of characteristics among groups according to age

According to the cutoff points identified by the X-tile program, the cohort of patients was classified by age into the following subgroups: ≤49 years (*n* = 360), 50-69 years (*n* = 289), and ≥70 years (*n* = 77) (Table 2). The proportion of races in each group was comparable with slight variations (Table 2). The proportion of patients receiving extended surgery (TT or near-TT) gradually decreased with age (*p* = 0.025), but the application of beam radiation increased with age significantly (*p* = 0.006) when compared with the use of radioisotope treatment (*p* = 0.056) among the three groups. The proportion of conventional risk factors, such as male sex and T3/4 primary tumor, had an increasing trend with age; however, the incidences of local node metastasis (N stage, *p* = 0.675) and distant metastasis (M stage, *p* = 0.056) were comparable among subgroups based on age. Finally, the overall pathologic TNM staging indicated that the elderly group (≥70 years) had the highest proportion of patients with advanced MTC (Stage IV, 53.2%) followed by the 50-69 years age subgroup (Stage IV, 49.1%); the relatively younger group (≤ 49 years) had obviously less patients with Stage IV MTC (12.8%). All the differences in proportions among age subgroups were significant (*p* = 0.001).

**Table 2:** Comparisons of clinicopathological characteristics of MTC patients from the SEER database by age subgroups

Variable	≤ 49 years	50–69 years	≥ 70 years	*P* value
	(n=360)	(n=289)	(n=77)	
Sex				
Male	134 (37.2)	130 (45.0)	39 (50.6)	0.034
Female	226 (62.8)	159 (55.0)	38 (49.4)	
Race				
Black	33 (9.2)	30 (10.4)	3 (3.9)	0.370
White	308 (85.6)	249 (86.2)	70 (90.9)	
T stage				
T1/T2	234 (65.0)	174 (60.2)	37 (48.1)	0.019
T3/T4	126 (35.0)	115 (39.8)	40 (51.9)	
N stage				
N0	168 (46.7)	122 (42.2)	30 (39.0)	0.675
N1a	55 (15.3)	45 (15.6)	13 (16.9)	
N1b	137 (38.1)	122 (42.2)	34 (44.2)	
M stage				
M0	322 (89.4)	241 (83.4)	64 (83.1)	0.056
M1	38 (10.6)	48 (16.6)	13 (16.9)	
TNM stage				
I	261 (72.5)	65 (22.5)	16 (20.8)	0.001
II	37 (10.3)	36 (12.5)	8 (10.4)	
III	16 (4.4)	46 (15.9)	12 (15.6)	
IV	46 (12.8)	142 (49.1)	41 (53.2)	
Surgical treatment				
Less than TT	20 (5.6)	14 (4.8)	10 (13.0)	0.025
TT or near TT	340 (94.4)	275 (95.2)	67 (87.0)	
Postoperative treatment				
Radioisotopes	12 (3.3)	8 (2.8)	2 (2.6)	0.056
Beam radiation	39 (10.8)	47 (16.3)	21 (27.3)	0.006

Data are presented as n (%).

Group comparisons of categorical variables were performed using the chi-square test or Fisher's exact test for small cell values.

Abbreviations: MTC, medullary thyroid cancer; SEER, Surveillance, Epidemiology, and End Results; TT, total thyroidectomy.

### Overall impact of age on survival in MTC and subgroup analysis

Although MTC is a relatively indolent malignancy, with reported 10-year survival rates from 69% to 89% [12, 13], we observed the worsening of CSS rates with increasing age by Kaplan-Meier curves from the X-tile plots shown in Figure 1. To determine how strongly increasing age was associated with mortality relative to other known risk factors of MTC, we performed multivariate Cox regression analysis (Table 3). The risk of cancer-specific mortality increased significantly in the 50-69 years age subgroup (HR 2.853, 95% CI 1.631-4.99) and ≥70 years age subgroup (HR 5.804, 95% CI 2.915-11.555) when compared to the ≤49 years age subgroup.

**Table 3:** Multivariate Cox regression for CSS in MTC patients from SEER database

Independent variable	Multivariate Analysis	
	HR (95% CI)	*P* value
Sex (Female *vs.* Male)	1.238 (0.770–1.990)	0.379
Age (years)		
≤ 49	1 (Reference)	
50-69	2.853 (1.631–4.992)	0.001
≥ 70	5.804 (2.915–11.555)	0.001
Race (White vs. Black)	0.344 (0.188–0.630)	0.001
T stage (T3/4 *vs.* T1/2)	3.931 (2.093–7.381)	0.001
N stage		
N0	1 (Reference)	
N1a	3.269 (1.386–7.710)	0.007
N1b	3.991 (1.548–10.292)	0.004
Distant metastasis	3.998 (2.419–6.606)	0.001

Abbreviations: MTC, medullary thyroid cancer; CSS, cancer-specific survival; SEER, Surveillance, Epidemiology, and End Results; HR, hazard ratio; CI, confidence interval.

**Figure 2:**
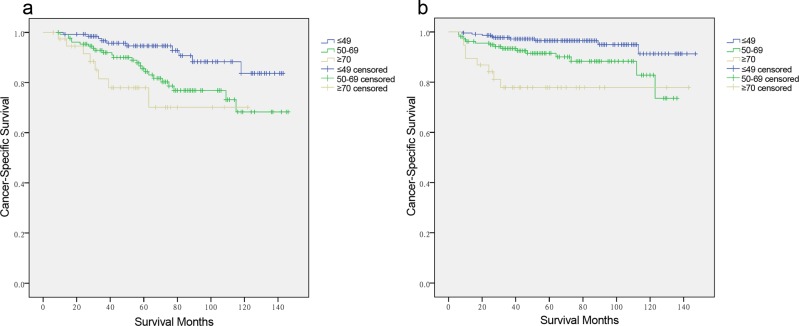
Log-rank tests of cancer-specific survival comparing among ≤ 49, 50-69, and ≥70 years age subgroups for **a.** males: χ^2^ = 11.586, *p* = 0.003. **b.** females: χ^2^ = 18.907, *p* = 0.001.

**Figure 3:**
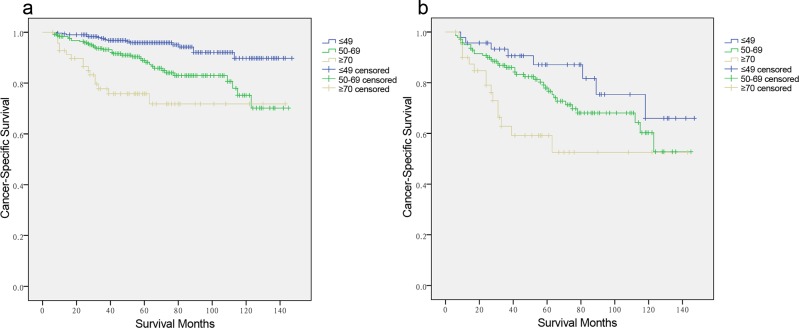
Log-rank tests of cancer-specific survival comparing among ≤ 49, 50-69, and ≥70 years age subgroups for **a.** non-Hispanic White: χ^2^ = 0.315, *p* = 0.003. **b.** stage IV: χ^2^ = 9.330, *p* = 0.009.

Results from the Cox proportional hazards regression models identified and verified a series of risk predictors for mortality in MTC, including race and TNM stage, which were consistent with previous studies [14, 15]. One reason for the poor prognosis in elderly patients is associated with these factors at diagnosis. For example, the elderly group (≥70 years) had the highest proportion of Stage IV patients among the age subgroups. To investigate the influence of age itself by minimizing interference from conventional risk factors, we conducted subgroup analysis for the effects of age on survival in MTC stratified by sex, race, and TNM stage. On proportional hazards regression models, elderly age (≥70 years) was associated with poorer survival in each sex subgroup by Kaplan-Meier curves (in males, Figure 2a, χ^2^ log-rank = 11.586, *p* = 0.003; in females, Figure 2b, χ^2^ log-rank = 18.907, *p* = 0.001); but this association was only significant in females after being adjusted for race and TNM stage (Table 4, ≤49 years as reference; 50-69 years, HR 1.853, 95% CI 1.363-2.004; ≥70 years, HR 2.992, 95% CI 1.109-8.076). Increasing age predicted high risk of mortality only in non-Hispanic white patients in both univariate (Figure 3a, χ^2^ log-rank = 0.315, *p* = 0.001) and multivariate analyses (Table 4, ≤49 years as reference; 50-69 years, HR 1.722, 95% CI 1.483-1.912; ≥70 years, HR 2.208, 95% CI 1.062-4.592). According to the TNM stage, the prediction of age on poorer CSS was only significant in Stage IV stratification, according to both univariate (Figure 3b, χ^2^ log-rank = 9.330, *p* = 0.009) and multivariate analyses (Table 4, ≤49 years as reference; 50-69 years, HR 1.697, 95% CI 1.387-3.660; ≥70 years, HR 3.763, 95% CI 1.565-9.050).

**Table 4:** Multivariate analysis of age on CSS in MTC according to clinicopathological variables

	Multivariate Analysis	
Variable per age subgroup	HR (95% CI)	*P* value
Sex^1^		
Male		
≤ 49 years	1 (Reference)	
50–69 years	1.079 (0.497–2.342)	0.847
≥ 70 years	1.738 (0.656–4.608)	0.267
Female		
≤ 49 years	1 (Reference)	
50–69 years	1.853 (1.363–2.004)	0.015
≥ 70 years	2.992 (1.109–8.076)	0.030
Race^2^		
African American		
≤ 49 years	1 (Reference)	
50–69 years	1.545 (0.350–6.823)	0.566
≥ 70 years	None	0.989
Non-Hispanic white		
≤ 49 years	1 (Reference)	
50–69 years	1.722 (1.483–1.912)	0.046
≥ 70 years	2.208 (1.062–4.592)	0.034
TNM stage^3^		
Stage I		
≤ 49 years	1 (Reference)	
50–69 years	None	0.978
≥ 70 years	None	0.990
Stage II		
≤ 49 years	1 (Reference)	
50–69 years	0.190 (0.022–1.644)	0.131
≥ 70 years	None	0.990
Stage III		
≤ 49 years	1 (Reference)	
50–69 years	None	0.966
≥ 70 years	None	0.962
Stage IV		
≤ 49 years	1 (Reference)	
50–69 years	1.697 (1.387–3.660)	0.018
≥ 70 years	3.763 (1.565–9.050)	0.003

1 *P*-value for HR was adjusted for race and TNM stage as covariates.

2 *P*-value for HR was adjusted for sex and TNM stage as covariates.

3 *P*-value for HR was adjusted for sex and age as covariates.

None refers to lack of results because of analysis incapability.

Abbreviations: MTC, medullary thyroid cancer; CSS, cancer-specific survival; HR, hazard ratio; CI, confidence interval; TNM, tumor type, lymph node involvement, distant metastasis.

### Prognostic nomogram for CSS and comparison of predictive accuracy for CSS between the nomogram and conventional staging system

**Figure 4:**
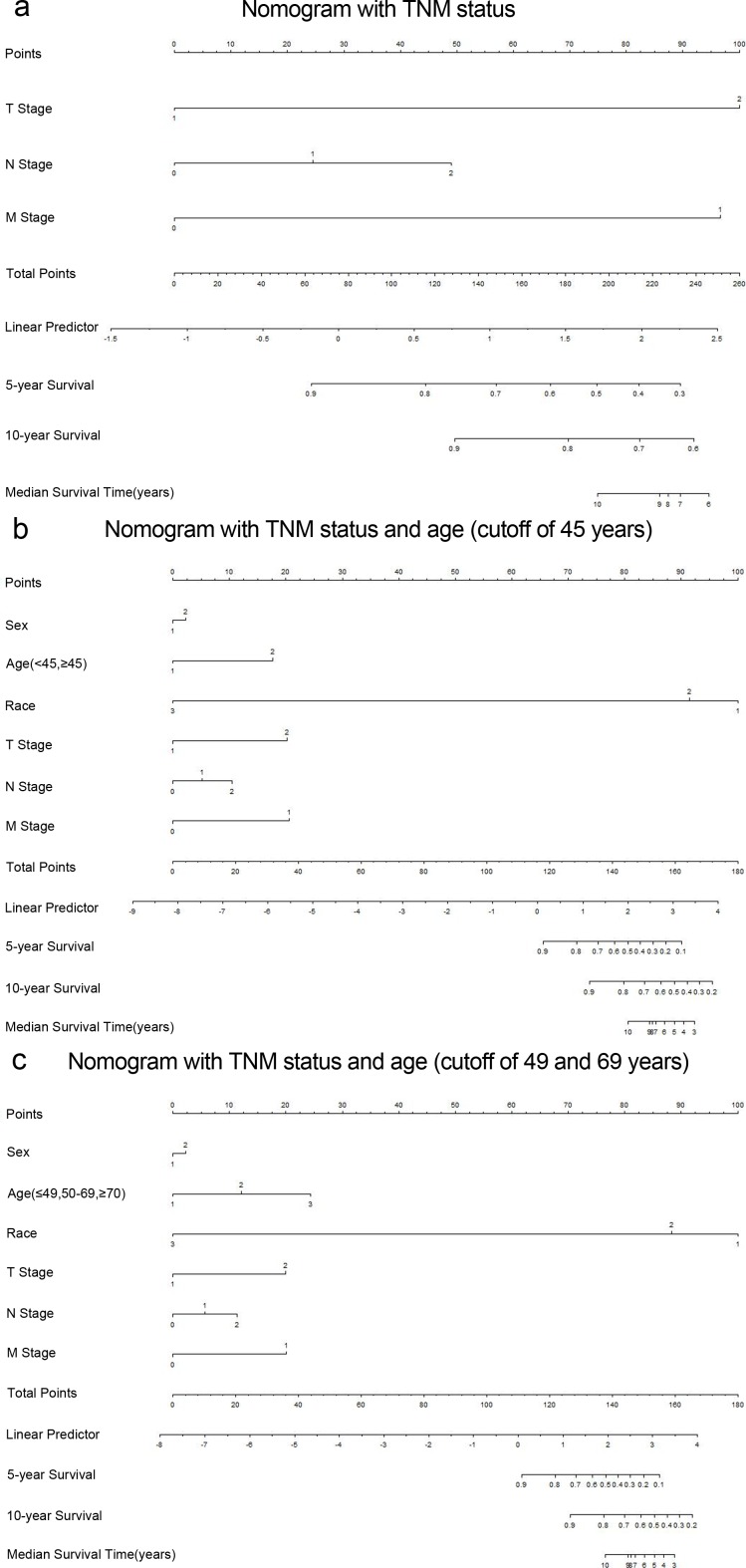
Medullary thyroid cancer-specific survival Nomograms (To use the nomogram, an individual patient's value is located on each variable axis, and a line is drawn upward to determine the number of points received for each variable value The sum of these numbers is located on the Total Points axis, and a line is drawn downward to the survival axes to determine the likelihood of 5- or 10- year survival). **a.** T stage 1, T1/2; T stage 2, T3/4; N stage 1, N1a; N stage 2, N1b; M stage 1, distant metastasis. **b.** Sex 1, male; Sex 2, female; Race 1, black; Race 2, non-Hispanic White; Race 3, others; Age 1, < 45 years; Age 2, ≥45 years. **c.** Age 1, < 49 years; Age 2, 50-69 years; Age 3, ≥70 years.

**Figure 5:**
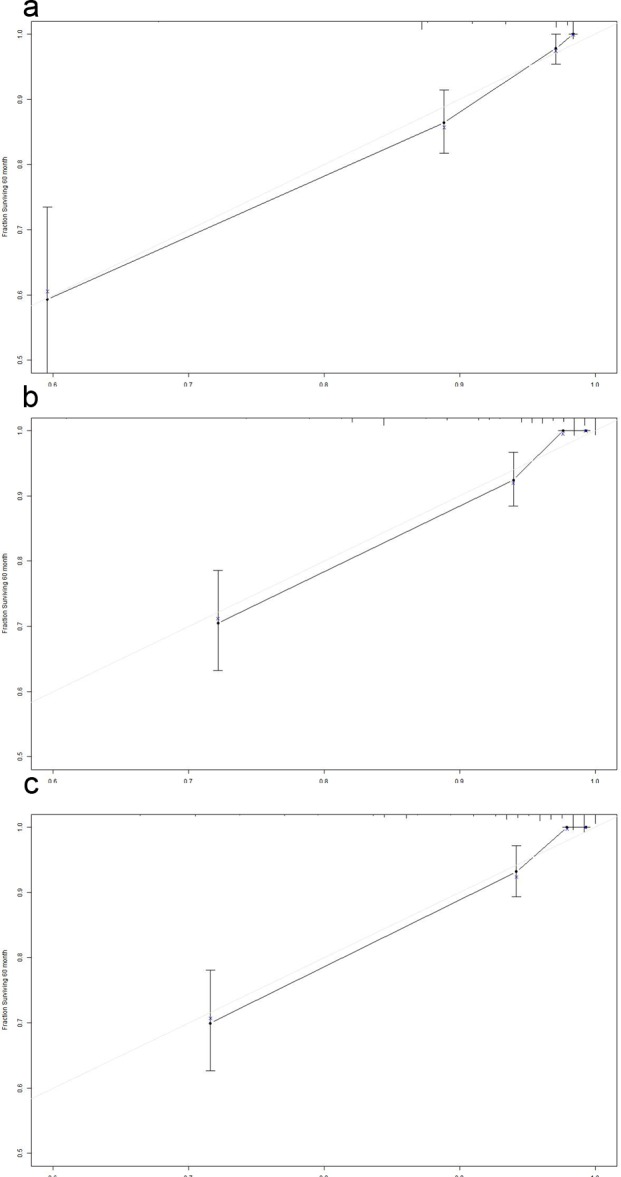
The calibration curve for predicting patient survival at 5 years in the primary cohort **a.** TNM. **b.** Age (cutoff of 45 years)/Sex/Race/TNM. **c.** Age (cutoff of 49 and 69 years) /Sex/Race/TNM. Nomogram-predicted probability of overall survival is plotted on the x-axis; actual overall survival is plotted on the y-axis.

To our knowledge, the new TNM classification is one of the most commonly used systems for cancer staging that adopted an age cutoff of 45 years to stratify the patients in high- and low-risk groups for cancer-specific mortality in DTC, but not for MTC. To predict the CSS of patients with MTC, age (three categories with cutoffs of 49 and 69 years, or binary classification with the conventional cutoff of 45 years), sex, race, and pathologic TNM status were selected for the final model as having the highest predictive accuracy for risk for cancer-specific mortality[16] (Figure 4a, 4b and 4C). The C-index for CSS prediction with TNM, age (cutoff of 45 years)/sex/race/TNM and age (cutoff of 49 and 69 years)/sex/race/TNM were 0.832 (95% CI 0.763-0.901), 0.863 (95% CI 0.799-0.928), and 0.876 (95% CI 0.817-0.935), respectively (Figure 5a, 5b and 5c). The calibration plot for the probability of survival at 5 or 10 years after surgery showed an optimal agreement between the prediction by the nomogram and actual observations.

## DISCUSSION

Because of the low incidence of MTC and lack of prospective studies evaluating the prognosis of patients with MTC, there is still a lack of convincing evidence to show that additional risk factors, other than TNM factors, could predict long-term outcomes and improve the predictive accuracy of the current staging system. A recent retrospective single-center study reviewing of 104 patients with MTC, suggested that age and stage were prognostic indicators by multivariate analysis [17]. The purpose of this population-based cohort study was to evaluate whether age could be an optimal predictive factor of clinical outcomes in MTC. We also have designed a model that systematically considers multiple variables and examined the superiority of age to conventional staging based on the cause-specific mortality of each MTC patient.

A total of 725 patients with MTC were selected from the SEER cancer database. We found that age at diagnosis was a significant predictive factor of death from cancer. We then used the X-tile program to identify 49 and 69 as cutoff values in terms of survival, with the minimum *p* values from log-rank χ^2^ statistics, which made our results more convincing. We then adopted age-based categorical variables to risk-stratify patients in multivariate analysis. The results confirmed that age was a significant predictor for CSS in the entire MTC cohort (50-69 years are subgroup, HR 2.853, 95% CI 1.631-4.99; ≥70 years are subgroup, HR 5.804, 95% CI 2.915-11.555), especially among female patients, non-Hispanic white patients, and those with stage IV disease. Thus, the predictive accuracy for patients with MTC who undergo surgery might correlate with these factors. The models including multiple variables based on conventional TNM staging systems to predict the cause-specific mortality of each MTC patient were observed to have C-indices ranging from 0.832 to 0.876 for survival prediction in the primary cohort. The highest C-index (0.876) indicated that the nomogram constructed by the four categories (age, sex, race and TNM status) performed well in predicting survival compared with the widely used TNM staging system in MTC patients.

To illustrate the impact of age and utility of the nomogram designed for this study, we present a hypothetical patient with MTC. A 71-year old non-Hispanic white female with a T3N1M0 MTC has a 75.6% of 10-year CSS predicted by the three age subgroups in the nomogram and an 87.1% 10-year CSS predicted by binary age nomogram; however, the 7th edition of the AJCC TNM staging system indicated that this patient had a 90.6% 10-year CSS, which is a better prognosis than either one from our nomograms.

It is important to mention that our study has limitations. First, the nomogram was developed from the collection of retrospective data of MTC cases from the SEER database, making it difficult to compare sporadic and hereditary MTC. Sporadic MTC tends to be more aggressive than hereditary MTCs, and they frequently metastasize to cervical LNs. This may impact on the predictive value of age between the two clinical settings. Second, the SEER database lacks information on the measurement of calcitonin, which is produced by thyroid C cells and MTC cells. The measurement of calcitonin is helpful in screening patients at risk for MTC as well as in their follow-up after treatment. Finally, whether this nomogram could be applied to patients who receive treatment remains to be determined in the future.

Overall, our analysis of the SEER database revealed that patient age at the time of MTC diagnosis is an independent risk factor with adverse impact on the CSS. Using the cutoff points 49 and 69, the MTC patients were classified into three risk groups. The age-stratification can be used together with sex, race and conventional TNM status to select high-risk patients for cancer-specific mortality. This designed staging system may be of greater prognostic value compared with the sole AJCC TNM staging classification, which may be useful for patient counseling in terms of prognosis and subsequent clinical follow-up.

## MATERIALS AND METHODS

### Patient selection from the SEER database

We extracted data from the SEER cancer registry. The SEER, a population-based registry sponsored by the National Cancer Institute, collects information on cancer incidence and survival from 17 population-based cancer registries, including approximately 28% of the US population [18]. The SEER data contain no identifiers and is publicly available for use in studies on cancer-based epidemiology and health policy. The National Cancer Institute's SEER*Stat software (www.seer.cancer.gov/seerstat) (Version 8.1.2) was used to identify patients who were diagnosed with single primary MTC between 2000 and 2010. Patients with thyroidectomy, lymph node dissection and postoperative therapy for MTC were included. Histology types were limited to MTC (8510). Patients were excluded if their records held insufficient data or unknown clinicopathological profile, undetermined histology or other types of thyroid cancer.

### Ethics statement

This study was conducted in accordance with the Helsinki Declaration. An independent ethics committee/institutional review board at Fudan University Shanghai Cancer Center approved our study. Data released from the SEER database does not require informed patient consent because it contains no identifiers and is publicly available. We obtained permission to access the research data files in the SEER program by the National Cancer Institute, USA (reference number 10817-Nov2013).

### Clinicopathological variable assessment

Age, sex, race, surgical procedures, adjuvant therapies, TNM stage, and survival time were extracted from the SEER database. Race was categorized into African American, non-Hispanic white, and others (American Indian/AK Native, Asian/Pacific Islander) as provided by the SEER database. We followed the guidance of the 2010 TNM classification of the American Joint Committee on Cancer (AJCC) [19]. The endpoint was calculated from the date of diagnosis to the date of cancer-specific mortality and was shown as “SEER CSS” in the database.

### Statistical analysis

The Chi-square (χ^2^) test was used to compare patient baseline characteristics. Survival rate was generated using Kaplan-Meier curves, and the differences were compared with the log-rank test. The cutoff points for age ranges were analyzed using the X-tile program (http://www.tissuearray.org/rimmlab/)[20]. A Cox proportional hazards regression model was then built to evaluate the risks of variables on cancer mortality in MTC patients. The hazard ratio (HR) for relationships between variables and cancer-specific mortality was calculated using a binary Cox regression model. All confidence intervals (CIs) were stated at the 95% confidence level.

A nomogram was formulated based on the results of multivariate analysis and using the package of rms in R version 2.14.1 (http://www.r-project.org/). A final model selection was performed by a backward stepdown selection process with the Akaike information criterion. The performance of the nomogram was measured by the concordance index (C-index) and assessed by comparing nomogram-predicted versus observed Kaplan-Meier estimates of survival probability [21, 22]. The C-index and calibration curve were derived based on the regression analysis. All *p* values were 2-sided. *P* < 0.05 was considered statistically significant.

## Abbreviations

MTC: medullary thyroid cancer
CSS: cancer-specific survival
HR: hazard ratio
CI: confidence interval
TNM: tumor type, lymph node involvement, distant metastasis.
